# Impact of aminoglycosides on survival rate and renal outcomes in patients with urosepsis: a multicenter retrospective study

**DOI:** 10.1186/s13613-025-01469-5

**Published:** 2025-04-12

**Authors:** David Rozenblat, Arnaud Serret-Larmande, Alexis Maillard, Romain Arrestier, Sarah Benghanem, Julien Charpentier, Michael Darmon, Vincent Das, François Dépret, Jean Luc Donay, Hervé Jacquier, Hélène Poupet, Jean-Michel Molina, Matthieu Lafaurie

**Affiliations:** 1 Service de Maladies infectieuses et Tropicales, Hôpitaux Universitaires Saint-Louis Lariboisière, Assistance Publique - Hôpitaux de Paris, 75010 Paris, France; 2 Service de Biostatistiques et Information Médicale, Hôpitaux Universitaires Saint-Louis Lariboisière, Assistance Publique - Hôpitaux de Paris, 75010 Paris, France; 3 Service de Médecine Intensive Réanimation, Hôpitaux Universitaires Saint-Louis Lariboisière, Assistance Publique - Hôpitaux de Paris, 75010 Paris, France; 4 Service de Médecine Intensive Réanimation, Hôpitaux Universitaires Henri Mondor, Assistance Publique - Hôpitaux de Paris, 94010 Créteil, France; 5 Service de Médecine Intensive Réanimation, Hôpitaux Universitaire Paris Centre, Hôpital Cochin, Assistance Publique - Hôpitaux de Paris, 75014 Paris, France; 6 Service de Médecine Intensive Réanimation, Centre Hospitalier Intercommunal André Grégoire, Groupe Hospitalier de Territoire Grand Paris Nord-Est, 93100 Montreuil, France; 7 Service d’Anesthésie-Réanimation et traitement chirurgical des grands brûlés, Hôpitaux Universitaires Saint-Louis Lariboisière, Assistance Publique - Hôpitaux de Paris, 75010 Paris, France; 8 Service de Bactériologie, Hôpitaux Universitaires Saint-Louis Lariboisière, Assistance Publique - Hôpitaux de Paris, 75010 Paris, France; 9 Service de Bactériologie, Hôpitaux Universitaires Henri Mondor, Assistance Publique - Hôpitaux de Paris, 94010 Créteil, France; 10 Service de Bactériologie, Hôpitaux Universitaire Paris Centre, Hôpital Cochin, Assistance Publique - Hôpitaux de Paris, 75014 Paris, France; 11 Sorbonne Université, Paris, France; 12 Université Paris Cité, Paris, France; 13 INSERM, Institut Pierre Louis d’Épidémiologie et de Santé Publique, AP-HP, Hôpital Pitié-Salpêtrière, Département de Santé Publique, Centre de Pharmaco-épidémiologie, Sorbonne Université, Paris, France

**Keywords:** Urinary tract infections, Septic shock, Aminoglycosides, Acute kidney injury, Mortality

## Abstract

**Background:**

Combination therapy with a beta-lactam and an aminoglycoside is currently recommended for the empirical treatment of urosepsis. Nephrotoxicity is the most common adverse effect of aminoglycosides and acute kidney injury (AKI) has a significant prognostic impact in septic shock. This study aimed to evaluate the impact of empirical antibiotic therapy with or without an aminoglycoside on survival and renal outcomes in patients admitted to the intensive care unit (ICU) with urosepsis.

**Methods:**

This multicenter, retrospective, comparative study included all adults admitted to the ICU for urinary sepsis or septic shock between January 2015 and May 2022 in four ICUs of three university hospitals within the Assistance Publique—Hôpitaux de Paris (APHP). The primary outcome was mortality on day 30 after ICU admission. Secondary endpoints included the lack of renal recovery, the need for new renal replacement therapy (RRT), the Major Adverse Kidney Events at day 30 (MAKE 30) and ICU length of stay. Confounding by indication was taken into account using propensity score weighting.

**Results:**

A total of 580 patients were included, median age was 69 years (interquartile: 58–77) and 53.6% were male. Overall, 335 patients (57.8%) were in septic shock and 448 (79.2%) had AKI on admission. A total of 579 patients (99.8%) received a beta-lactam as empirical therapy (with (n = 444) or without (n = 136) aminoglycosides). The overall 30-day mortality rate was 10.5% (61/580). After propensity score weighting, the mortality rate in patients receiving aminoglycosides was 7.7% (7/91) compared to 12.1% (11/91) in those not receiving aminoglycosides (adjusted hazard ratio (aHR) = 0.65 [0.35; 1.23], *p* = 0.19). No significant differences were found in the lack of renal recovery at day 30 (aHR = 0.88 [0.49; 1.58], *p* = 0.67), the need for new RRT within 30 days (aHR = 1.01 [0.54; 1.88], *p* = 0.97), MAKE 30 (aHR = 0.94 [0.60; 1.50], *p* = 0.81), and ICU length of stay among survivors (aHR = 1.07 [0.87; 1.31], *p* = 0.53).

**Conclusions:**

Including aminoglycosides in the empirical antibiotic therapy did not significantly improve 30-day survival in patients admitted to the ICU for urosepsis. However, the use of aminoglycosides was not associated with worse renal outcomes.

**Supplementary Information:**

The online version contains supplementary material available at 10.1186/s13613-025-01469-5.

## Background

Urosepsis, defined as sepsis caused by urinary tract infection, is the third most common cause of sepsis and septic shock in the intensive care unit (ICU), with a mortality rate reaching 13.5% [1–5]. Current American and European recommendations suggest the use of a combination therapy for the empirical treatment of urosepsis, while French guidelines specify the use of a combination of beta-lactams and aminoglycosides [6–8]. In a recent international survey, aminoglycosides were widely used in critically ill patients with urosepsis [9]. Several properties of aminoglycosides justify this use and recommendation: a broader antibacterial spectrum, rapid bactericidal activity, prolonged post-antibiotic effect, high intrarenal concentration and potential synergy with beta-lactams [10, 11]. Although this combination appears to be an attractive therapeutic option, its actual benefit remains uncertain. Animal modelling studies suggest a benefit of this combination, but clinical evidence of improved outcomes is scarce and based on controversial and retrospective studies [12–18]. Furthermore, none of these studies were conducted exclusively in patients with urosepsis, which may have confounded the conclusions.

Nephrotoxicity is one of the most important adverse effects of aminoglycosides. This toxicity is primarily tubular and can increase the risk of acute kidney injury (AKI), depending on the target population [19, 20]. In septic shock, the incidence and prognostic impact of AKI are well established, with an associated mortality rate of up to 60% [21–25]. In urinary septic shock, the incidence of AKI is particularly high due to the acute interstitial nephritis resulting from the infection which can reach 86% of cases [5]. Given the significant risk of AKI in patients with urosepsis and the associated mortality, additional evidence supporting the use of aminoglycosides in the treatment of urosepsis is a major challenge.

The aim of this study was to investigate the effect of empiric antibiotic therapy with or without an aminoglycoside on mortality and renal outcomes in patients admitted to the ICU with urosepsis.

## Methods

### Design and population

The AMINURO study was a multicenter, retrospective study in four ICUs of three university hospitals within the Assistance Publique—Hôpitaux de Paris (APHP). The study included all patients over 18 years of age admitted to the ICU with urosepsis (urinary sepsis or septic shock) between January 2015 and May 2022. Sepsis was defined according to the SEPSIS III criteria and septic shock was characterized by the need for vasopressors [26]. Urinary origin was confirmed by isolation of bacteria from urine cultures (≥ 10^3^ CFU/mL), with or without concurrent positive blood cultures with the same organism(s), and was considered causative for the sepsis by the attending clinician [7]. Patients with suspected urosepsis without isolation of bacteria from urine samples were excluded. This study was approved by the Research Ethics Committee for Infectious and Tropical Diseases (CER-MIT-2023-0503) in Paris, France, and was conducted in accordance with the tenets of the Declaration of Helsinki.

### Data collection and definitions

Patients were identified in the APHP digital data system using the following diagnostic terms: “pyelonephritis”, “acute tubulointerstitial nephritis”, “pyonephrosis”, "kidney abscess”, “urinary tract infection”, “acute prostatitis”, “prostate abscess”, “prostato-cystitis” and “septic shock”. Data were collected retrospectively from electronic medical records and included information on demographics, comorbidities, current medications, laboratory values, microbiological findings (bacterial cultures and antibiotic susceptibility testing), organ support therapies, Charlson Comorbidity Index, Sequential Organ Failure Assessment (SOFA) score and Simplified Acute Physiology Score II (SAPS II). Healthcare-associated infections (HCAI) were defined as infections detected on hospital admission or within 48 h thereafter that met at least one of the following criteria: hospitalization in an acute care or rehabilitation facility for 2 or more days in the 90 days prior to the current hospitalization, antibiotic treatment in the 90 days prior to the current episode, an invasive urinary procedure in the 30 days prior to the current episode, the presence of a long-term indwelling urethral catheter or nephrostomy, residence in a nursing home or long-term care facility, or chronic dialysis. Community-acquired infections (CAI) were defined as infections detected at hospital admission or within the first 48 h without meeting any of the criteria for HCAI. Infections detected more than 48 h after hospital admission were considered hospital-acquired infections (HAI) [27]. Chronic kidney disease was defined as a glomerular filtration rate (GFR) of less than 60 mL/min/1.73 m^2^ for more than 3 months [28]. Congestive heart failure (CHF) was identified according to the Charlson Comorbidity Index. Neutropenia was defined as a neutrophil count of less than 0.5 G/L. Stages of AKI were classified using the Kidney Disease Improving Global Outcomes (KDIGO) criteria for serum creatinine (SCr) level (stage 1: increase in SCr from 1.5 to 1.9 times baseline or increase ≥ 26.5 μmol/L; stage 2: increase in SCr from 2.0 to 2.9 times baseline; stage 3: increase in SCr from 3.0 times baseline or increase in ≥ 353.6 μmol/L or initiation of renal replacement therapy (RRT)) [28]. Previous renal function was estimated using baseline SCr, defined as the most recent value available before ICU admission in the past year. If this value was not available and the patients had no history of chronic kidney disease (CKD), baseline SCr was estimated using the lowest SCr between the SCr on hospital admission and the SCr back-calculated from the CKD-EPI equation assuming a GFR of 75 mL/min/1.73 m^2^, according to current recommendations [28–30]. Empirical antibiotic therapy was defined as the initiation of antibiotics before identification of the infecting microorganism and its antibiotic susceptibility. Combination therapy with aminoglycosides was defined as the concomitant use of at least two classes of antibiotics, including an aminoglycoside administered between 24 h before and up to 72 h after admission to the ICU. All patients who received at least one injection of an aminoglycoside, regardless of dose, were included in the aminoglycoside group.

### Outcomes

The primary outcome was mortality on day 30 after ICU admission. Patients discharged alive from hospital before day 30 were considered alive on day 30. Secondary endpoints included: lack of renal recovery (only in patients who developed AKI), defined as a ratio of the last SCr (measured before day 30 or hospital discharge whichever came first) to baseline SCr greater than 200%, the need for new RRT within 30 days, and ICU length of stay among survivors (restricted to survivors to account for the competing risk of death) [31, 32]. Major adverse kidney events at day 30 (MAKE 30) were also assessed. MAKE 30 was defined as a composite of the following criteria, assessed 30 days after admission or at hospital discharge, whichever occurred first: death, new RRT, or lack of renal recovery as defined above [32].

### Statistical analysis

Variables were described using counts and proportions for categorical variables and medians and interquartile (IQR) for continuous variables. For univariate analyses, categorical variables were compared using the Chi-square test or Fisher’s exact test, as appropriate. Continuous variables were compared by ANOVA. Ordinal categorical variables were compared using the Cochran–Armitage test for trend. We employed propensity score weighting with overlap weights to account for potential confounding by indication [33]. Overlap weights target the population which mimics the characteristics of a pragmatic randomized trial. Moreover, they have been shown to overcome some of the limitations of other weighting formulas such as Inverse Probability of Treatment Weighting (IPTW), and optimize precision of the treatment effect estimate among propensity score weighting methods [34]. Henceforth, we will refer to this adjustment methodology of propensity score weighting with overlap weights simply as “overlap weighting”. The propensity score was estimated using a logistic regression model that included covariates that could influence the decision to use aminoglycosides and affect prognosis. These covariates were selected through a dual approach, combining clinical expertise with statistical analysis to ensure comprehensive confounding control [35]. Clinically meaningful covariates were chosen based on expert knowledge (infectiologists, intensivists and nephrologists), and included demographics, comorbidities, severity scores, admission characteristics, and microbiological characteristics. To complement this and capture potential confounders that may have been overlooked by clinical expertise, relevant variables that were associated with mortality in univariate analyses (*p* < 0.10) and were not highly correlated with variables already included based on clinical relevance were added to the propensity score model (Additional file 1: File 1). In all, the variables included in the propensity score model were: septic shock, urinary tract materials, diabetes, age > 65 years, modified SOFA score (defined as SOFA score without cardiovascular and renal criteria), kidney transplant, infection onset source, immunosuppression, neutropenia, bacteria susceptible to the beta-lactam used, acute kidney injury, Charlson comorbidity index, congestive heart failure, polymicrobial infection, positive blood culture, and previous cancer (Additional file 1: File 2). The post-weighting sample size will be referred to as the Effective Sample Size. Weighted Cox proportional hazards models were used to estimate the association between outcomes and aminoglycoside treatment. Overlap weighting validity assumptions were checked (Additional file 1: Files 3 and 4). Subgroup analyses were performed for septic shock, positive blood cultures, kidney transplant status, presence of third-generation cephalosporin-resistant (3GC-R) Enterobacterales and bacterial susceptibility to the beta-lactam used. All statistical analyses were performed using R software, version 4.1.1 (R Foundation for Statistical Computing, Vienna, Austria), with the survival package used for survival modelling. A *p*-value of less than 0.05 was considered statistically significant for all analyses.

## Results

### Study population

Of the 5283 patients identified in the four ICUs between January 2015 and May 2022, using the APHP digital data system, 580 with documented urosepsis were included in the study. The median age was 69 years (IQR: 58–77), and 53.6% of patients were male. Of these, 515 (88.8%) were admitted to the medical ICU, while 65 (11.2%) were admitted to the surgical ICU. CAI accounted for 36.2%, HCAI for 42.1% and HAI for 21.7% of the urosepsis cases. Overall, 335 patients (57.8%) presented with septic shock and 146 (25.2%) had urinary tract obstruction requiring urgent diversion. AKI was observed on admission in 448 patients (79.2%). Of those without AKI on admission, 21 patients (3.6%) developed AKI after a median of 2 days (IQR: 0–4). A total of 579 patients (99.8%) were empirically treated with a beta-lactam, 214 (36.9%) with a third-generation cephalosporin, 16 (2.8%) with cefepime, 176 (30.3%) with piperacillin-tazobactam and 175 (30.2%) with carbapenem. In all, 444 patients (76.6%) received a combination of antibiotics that included an aminoglycoside, while 136 (23.4%) received no aminoglycoside. Specifically, 380 (85.6%) patients received amikacin and 63 (14.2%) patients received gentamicin. Aminoglycosides were only used in combination as part of empirical treatment, and no patient received an aminoglycoside as definitive therapy. Aminoglycoside therapy was not monitored. The baseline characteristics of the patients are shown in Table 1 (Additional file 1: Files 5, 6 and 7). Before weighting, patients treated with aminoglycosides had higher severity of illness scores, were more frequently in septic shock and had a higher incidence of positive blood cultures. Applying overlap weights to the cohort resulted in an effective simple size weighted subpopulation of 91 patients per treatment group. The characteristics of this weighted population, which was used for all the subsequent analyses, are shown in Table 2 and Additional file 1: File 8.

**Table 1:** Baseline patient characteristics

Variables	Without AG (n = 136)	With AG (n = 444)	Total (n = 580)
Age (median, IQR)	69 (58–78)	69 (58–77)	69 (58–77)
Male	66 (48.5%)	245 (55.2%)	311 (53.6%)
Infection onset source			
Community-acquired	49 (36.0%)	161 (36.3%)	210 (36.2%)
Healthcare-associated	62 (45.6%)	182 (41.0%)	244 (42.1%)
Hospital-acquired	25 (18.4%)	101 (22.7%)	126 (21.7%)
Comorbidities			
Chronic kidney disease	38 (27.9%)	157 (35.4%)	195 (33.6%)
Hemodialysis	0 (0.0%)	14 (3.2%)	14 (2.4%)
Kidney transplant	13 (9.6%)	68 (15.3%)	81 (14.0%)
Uropathy^a^	32 (23.5%)	128 (28.8%)	160 (27.6%)
Diabetes	39 (28.7%)	144 (32.4%)	183 (31.6%)
Congestive heart failure	16 (11.8%)	60 (13.5%)	76 (13.1%)
Previous malignancy^b^	62 (45.6%)	185 (41.7%)	247 (42.6%)
Immunosuppression^c^	37 (27.2%)	154 (34.7%)	191 (32.9%)
Charlson comorbidity index (median, IQR)	6 (4–8)	5.5 (4–8)	6 (4–8)
Admission characteristics			
SAPS II score (median, IQR)	44 (33–54)	48 (37–61)	46 (36–60)
SOFA score (median, IQR)	5 (3–7)	7 (5–10)	7 (4–9)
Septic shock	44 (32.4%)	291 (65.5%)	335 (57.8%)
Acute kidney injury	105 (77.2%)	343/430 (79.8%)	448/566 (79.2%)
KDIGO stage 1	49 (46.7%)	149 (43.4%)	198 (44.2%)
KDIGO stage 2	29 (27.6%)	87 (25.4%)	116 (25.9%)
KDIGO stage 3	27 (25.7%)	107 (31.2%)	134 (29.9%)
Mechanical ventilation	17 (12.5%)	98 (22.1%)	115 (19.8%)
Neutropenia^d^	1 (0.7%)	19 (4.3%)	20 (3.4%)
Urgent urinary diversion^e^	34 (25.0%)	112 (25.2%)	146 (25.2%)
Microorganism			
Enterobacterales	113 (83.1%)	384 (86.5%)	497 (85.7%)
*Proteus mirabilis*	11 (8.1%)	16 (3.6%)	27 (4.7%)
*Escherichia coli*	78 (57.4%)	261 (58.8%)	339 (58.4%)
*Klebsiella spp*^f^*/Citrobacter koseri*	28 (20.6%)	96 (21.6%)	124 (21.4%)
Inducible AmpC Enterobacterales^g^	14 (10.3%)	38 (8.6%)	52 (9.0%)
3GC-R Enterobacterales	22 (16.2%)	75 (16.9%)	97 (16.7%)
*Enterococcus spp*	13 (9.6%)	62 (14.0%)	75 (12.9%)
*Pseudomonas aeruginosa*	11 (8.1%)	35 (7.9%)	46 (7.9%)
*Staphylococcus aureus*	6 (4.4%)	6 (1.4%)	12 (2.1%)
Other pathogens	4 (2.9%)	22 (5.0%)	26 (4.5%)
Polymicrobial	24 (17.6%)	76 (17.1%)	100 (17.2%)
Positive blood culture	64 (47.1%)	272 (61.3%)	336 (57.9%)
ESBL-producing Enterobacterales colonization/infection in the 6 prior months	23 (16.9%)	67 (15.1%)	90 (15.5%)
GNB resistant to the BL used	12/113 (10.6%)	28/362 (7.7%)	40/475 (8.4%)
GNB resistant to the AG used	–	23/325 (7.1%)	–
GNB susceptible to at least one antibiotic used	101/113 (89.4%)	354/362 (97.8%)	455/475 (95.8%)
Antibiotic therapy			
3GC	51 (37.5%)	163 (36.7%)	214 (36.9%)
Cefepime	7 (5.1%)	9 (2.0%)	16 (2.8%)
Piperacillin-tazobactam	45 (33.1%)	131 (29.5%)	176 (30.3%)
Carbapenems^h^	31 (22.8%)	144 (32.4%)	175 (30.2%)
Amikacin	–	380 (85.6%)	–
Gentamicin	–	63 (14.2%)	–
Vancomycin	9 (6.6%)	49 (11.0%)	58 (10.0%)
Other antibiotics^i^	19 (14.0%)	33 (7.4%)	52 (9.0%)

Values represent the “number of subjects (%)” except as noted; IQR: interquartile range

Missing data were present only for “GNB resistant to the BL used”, “GNB resistant to the AG used”, and “GNB susceptible to at least one antibiotic used” with denominators added in table cells when necessary to indicate the actual number available observations

AG, aminoglycosides; BL, beta-lactam; ESBL, extended spectrum beta-lactamase; GNB, Gram-negative bacteria; KDIGO, Kidney Disease Improving Global Outcomes; SAPS, Simplified Acute Physiology Score II; SOFA, Sequential Organ Failure Assessment; 3GC, third-generation cephalosporins; 3GC-R, third generation cephalosporin-resistant

^a^Uropathy: urethral catheter, ureteral stent, nephrostomy, ileal conduit, orthotopic neobladder

^b^Previous malignancy: previous cancer or hemopathy

^c^Immunosuppression: malignancy or autoimmune-related chemotherapy, ≥ 10 mg/day chronic prednisone equivalent

^d^Neutropenia: neutrophil count of < 500 cells/mm3

^e^Urgent urinary diversion: emergency ureteral stenting or nephrostomy

^f^Except *Klebsiella aerogenes*

^g^Inducible AmpC Enterobacterales: Enterobacterales with inducible chromosomal AmpC beta-lactamases

^h^Carbapenems: meropenem or imipenem

^i^Other antibiotics: amoxicillin/clavulanic acid (one patient), fluoroquinolone, spiramycin, clindamycin, daptomycin, linezolid and metronidazole

**Table 2:** Baseline patient characteristics after propensity score weighting

Variables	Without AG (n = 91)	With AG (n = 91)	Total (n = 182)	Absolute mean difference	Standardized mean difference
Age (median, IQR)	69 (58–79)	70 (59–77)	69 (58–78)	0.02	0.02
Male	42 (46.0%)	52 (57.2%)	94 (51.6%)	0.11	0.23
Infection onset source					0.05
Community-acquired	34 (37.1%)	34 (37.1%)	67 (37.1%)	0.00	
Healthcare-associated	40 (44.1%)	38 (42.2%)	78 (43.2%)	− 0.02	
Hospital-acquired	17 (18.7%)	19 (20.6%)	36 (19.7%)	0.02	
Comorbidities					
Chronic kidney disease	25 (27.3%)	29 (31.7%)	54 (29.5%)	0.04	0.10
Hemodialysis	0 (0.0%)	0 (0.0%)	0 (0.0%)	0.00	0.00
Kidney transplant	10 (10.8%)	10 (10.8%)	20 (10.8%)	0.00	0.00
Uropathy^a^	22 (24.8%)	24 (26.4%)	46 (25.6%)	0.02	0.04
Diabetes	28 (30.4%)	28 (30.4%)	55 (30.4%)	0.00	0.00
Congestive heart failure	11 (12.1%)	11 (12.1%)	22 (12.1%)	0.00	0.00
Previous malignancy^b^	40 (44.5%)	39 (43.1%)	80 (43.8%)	− 0.01	− 0.03
Immunosuppression^c^	26 (28.8%)	26 (28.8%)	53 (28.8%)	0.00	0.00
Charlson comorbidity index (median, IQR)	6 (4–7)	5 (4–8)	6 (4–8)	0.00	0.00
Admission characteristics					
SAPS II score (median, IQR)	45 (35–56)	44 (35–55)	44 (35–56)	− 0.09	− 0.09
SOFA score (median, IQR)	5 (3–7)	5 (4–8)	5 (3–8)	0.07	0.07
Septic shock	37 (40.9%)	37 (40.9%)	74 (40.9%)	0.00	0.00
Acute kidney injury	71 (78.4%)	71 (78.4%)	142 (78.4%)	0.00	0.00
KDIGO stage 1	32 (35.2%)	33 (36.8%)	65 (36.0%)	0.02	
KDIGO stage 2	21 (23.2%)	17 (18.3%)	38 (20.8%)	− 0.05	
KDIGO stage 3	18 (19.9%)	21 (23.3%)	39 (21.6%)	0.03	
Mechanical ventilation	13 (14.8%)	11 (12.3%)	25 (13.6%)	− 0.02	0.07
Neutropenia^d^	1 (1.1%)	1 (1.1%)	2 (1.1%)	0.00	0.00
Urgent urinary diversion^e^	25 (27.1%)	20 (21.9%)	44 (24.5%)	− 0.05	− 0.12
Microorganism					
Enterobacterales	75 (82.9%)	78 (85.4%)	153 (84.1%)	0.03	0.07
*Proteus mirabilis*	7 (7.9%)	2 (2.3%)	9 (5.1%)	− 0.06	− 0.24
*Escherichia coli*	53 (58.4%)	54 (60.1%)	107 (59.2%)	0.02	0.03
*Klebsiella spp*^f^*/Citrobacter koseri*	18 (19.7%)	18 (19.9%)	36 (19.8%)	0.00	0.01
Inducible AmpC Enterobacterales^g^	9 (10.1%)	8 (9.2%)	18 (9.7%)	− 0.01	− 0.03
3GC-R Enterobacterales	15 (16.4%)	15 (16.0%)	29 (16.2%)	− 0.01	− 0.01
*Enterococcus spp*	8 (8.9%)	13 (13.8%)	21 (11.3%)	0.05	0.15
*Pseudomonas aeruginosa*	7 (7.9%)	7 (8.0%)	14 (7.9%)	0.00	0.00
*Staphylococcus aureus*	4 (4.6%)	2 (2.2%)	6 (3.4%)	− 0.02	− 0.14
Other pathogens	3 (3.1%)	5 (5.3%)	8 (4.2%)	0.02	0.12
Polymicrobial	16 (17.1%)	16 (17.1%)	31 (17.1%)	0.00	0.00
Positive blood culture	46 (50.4%)	46 (50.4%)	92 (50.4%)	0.00	0.00
ESBL-producing Enterobacterales colonization/infection in the 6 prior months	14 (15.5%)	12 (12.9%)	26 (14.2%)	− 0.03	− 0.07
GNB resistant to the BL used	8/76 (10.6%)	7/75 (8.9%)	15/151 (9.8%)	− 0.02	− 0.06
GNB resistant to the AG used	–	5/67 (7.2%)	–	–	–
GNB susceptible to at least one antibiotic used	68/76 (89.4%)	73/75 (97.8%)	141/151 (93.5%)	0.08	0.35
Antibiotic therapy					
3GC	33 (36.5%)	41 (44.8%)	74 (40.6%)	0.08	0.17
Cefepime	4 (4.5%)	2 (2.5%)	6 (3.5%)	− 0.02	− 0.11
Piperacillin-tazobactam	31 (34.4%)	23 (25.7%)	55 (30.1%)	− 0.09	− 0.19
Carbapenems^h^	21 (22.8%)	25 (27.2%)	45 (25.0%)	0.04	0.10
Amikacin	–	75 (83.0%)	–	–	–
Gentamicin	–	15 (16.6%)	–	–	–
Vancomycin	7 (7.7%)	7 (8.0%)	14 (7.8%)	0.00	0.01
Other antibiotics	12 (13.1%)	7 (7.2%)	18 (10.2%)	− 0.07	− 0.20

Values represent the “number of subjects (%)” except as noted; IQR: interquartile range

Missing data were present only for “GNB resistant to the BL used”, “GNB resistant to the AG used”, and “GNB susceptible to at least one antibiotic used” with denominators added in table cells when necessary to indicate the actual number available observations

Reported patient numbers per treatment group represent the Effective Sample Size, rounded to the nearest integer. Actual Effective Sample Size per group is 90.74, rounded up to 91. Categories count may be decimal numbers because of the treatment groups resampling based on a weighting method. Displayed counts are rounded to the closest integer. Proportions are calculated based on the non-rounded values. Due to rounding, the total may differ by ± 1 from the sum using rounded counts from each group

AG, aminoglycosides; BL, beta-lactam; ESBL, extended spectrum beta-lactamase; GNB, Gram-negative bacteria; KDIGO, Kidney Disease Improving Global Outcomes; SAPS, Simplified Acute Physiology Score II; SOFA, Sequential Organ Failure Assessment; 3GC, third-generation cephalosporins; 3GC-R, third-generation cephalosporin-resistant

^a^Uropathy: urethral catheter, ureteral stent, nephrostomy, ileal conduit, orthotopic neobladder

^b^Previous malignancy: previous cancer or hemopathy

^c^Immunosuppression: malignancy or autoimmune-related chemotherapy, ≥ 10 mg/day chronic prednisone equivalent

^d^Neutropenia: neutrophil count of < 500 cells/mm3

^e^Urgent urinary diversion: emergency ureteral stenting or nephrostomy

^f^Except *Klebsiella aerogenes*

^g^Inducible AmpC Enterobacterales: Enterobacterales with inducible chromosomal AmpC beta-lactamases

^h^Carbapenems: meropenem or imipenem

### Microbiology

The most commonly identified pathogens were *Escherichia coli* (58.4%), *Klebsiella spp.* and *Citrobacter koseri* (21.4%), *Enterococcus* spp. (12.9%) and *Pseudomonas aeruginosa* (8.0%) (Table 1). In total, 336 (57.9%) patients had positive blood cultures and 97 patients (16.7%) were infected with 3GC-R Enterobacterales, of which 88 (15.2%) were extended-spectrum beta-lactamase (ESBL)-producing Enterobacterales. Among patients with community-acquired infections, 6.7% (14/210) were due to 3GC-R Enterobacterales, including 11 (5.2%) ESBL-producing Enterobacterales. In patients with known ESBL-producing Enterobacterales colonization, 43.3% (39/90) developed ESBL-producing Enterobacterales urosepsis. Among all Gram-negative bacteria (GNB), 8.4% (40/475) were resistant to the beta-lactam used empirically and 7.1% (23/325) to the aminoglycoside used (Table 1). In cases where GNB exhibited resistance to the beta-lactam administered, aminoglycosides remained effective in 71.4% (20/28). Of note, the aminoglycoside used in the 8 patients with urosepsis caused by GNB resistant to both beta-lactams and aminoglycosides was amikacin. Finally, when a carbapenem was administered, only one GNB isolate (*Pseudomonas aeruginosa*) exhibited carbapenem resistance (0.7%, 1/136).

### Clinical outcomes before propensity score weighting

Overall, 30-day mortality was 10.5% (61/580) and median ICU length of stay (among survivors) was 3 days (IQR: 2–5). In patients with septic shock, the 30-day mortality rate was 14.0% (47/335). Before propensity score weighting, the mortality rate was 10.4% in patients receiving aminoglycosides *versus* 11.0% in those not receiving aminoglycosides, with no statistically significant difference between groups (hazard ratio (HR) = 0.88 [0.49; 1.58], *p* = 0.77) (Additional file 1: File 9). Regarding patient subgroups, the 30-day mortality according to whether they received an aminoglycoside or not is shown in Table 3.

**Table 3:** Mortality at day 30 with and without aminoglycosides in patient subgroups

Subgroups	Death without AG (n = 15/136)	Death with AG (n = 46/444)
Septic shock (n = 335)	8/44 (18.2%)	39/291 (13.4%)
Positive blood culture (n = 336)	10/64 (15.6%)	32/272 (11.8%)
Kidney transplant (n = 81)	0/13 (0.0%)	7/68 (10.3%)
3GC-R Enterobacterales (n = 97)	2/22 (9.1%)	6/75 (8.0%)
Gram negative bacteria susceptible to the beta-lactam used (n = 435)	12/101 (11.9%)	32/334 (9.6%)
Gram negative bacteria resistant to the beta-lactam used (n = 40)	1/12 (8.3%)	1/28 (3.6%)

AG, aminoglycosides; 3GC-R, third-generation cephalosporin-resistant

### Clinical outcomes after propensity score weighting

**Fig. 1:**
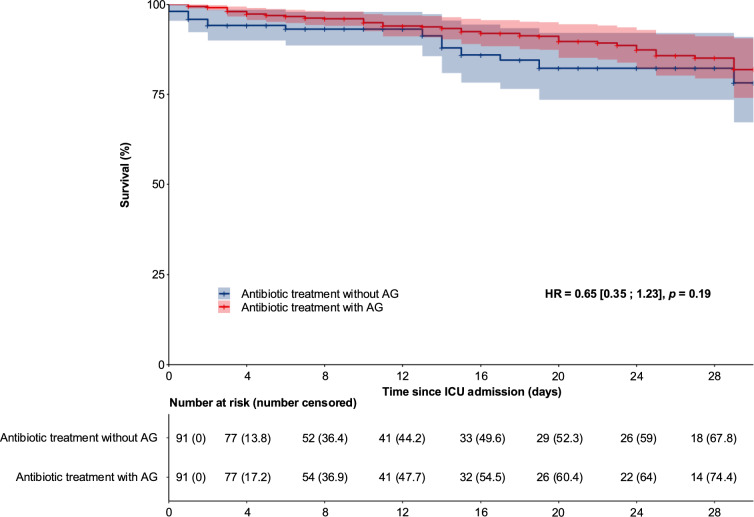
Overall survival with and without aminoglycosides after propensity score weighting. Kaplan–Meier curves showing the probability of survival according to the empirical antibiotic therapy group (treatment with aminoglycosides (AG) compared to treatment without AG) after propensity score weighting. The *p*-value was obtained from the Cox Proportional-Hazards Model

After propensity score weighting, the mortality rate was 7.7% in patients receiving aminoglycosides *versus* 12.1% in those not receiving aminoglycosides, with no statistically significant difference between groups (adjusted hazard ratio (aHR) = 0.65 [0.35; 1.23], *p* = 0.19) (Fig. 1). Regarding the secondary endpoints, there was no significant difference in the rate of no renal recovery (aHR = 0.88 [0.49; 1.58], *p* = 0.67), the need for new RRT (aHR = 1.01 [0.54; 1.88], *p* = 0.97), and the MAKE 30 criteria (aHR = 0.94 [0.60; 1.50], *p* = 0.81) (Table 4). ICU length of stay among survivors was not significantly different between the aminoglycoside and non-aminoglycoside groups (aHR = 1.07 [0.87; 1.31], *p* = 0.53). A sensitivity analysis using the Desirability of Outcome Ranking (DOOR) approach was performed [36]. This combined death, need for new RRT within 30 days, and lack of renal recovery at day 30 hierarchically ordered. The propensity score weighted Wilcoxon rank-sum test yielded *p* = 0.47, consistent with the main analysis findings. A multivariable Cox proportional hazards model assessing the association between aminoglycosides and mortality on day 30 was fitted, including the same covariates as those used in the propensity score model. The resulting adjusted HR was 0.50 [0.30; 1.17], *p* = 0.15, consistent with the results of the propensity score-weighted model.

**Table 4:** Outcomes in the propensity score-weighted population

Outcomes	Without AG (n = 91)	With AG (n = 91)	Total (n = 182)	Ajusted hazard ratio (95% CI)	*p*-value
Primary outcome					
Mortality at day 30	11 (12.1%)	7 (7.7%)	18 (9.9%)	0.65 (0.35; 1.23)	0.19
Secondary outcomes					
Lack of renal recovery at day 30*	12 (16.7%)*	10 (13.6%)*	22 (15.2%)*	0.88 (0.49; 1.56)	0.65
Need for RRT within 30 days	11 (12.1%)	10 (10.9%)	21 (11.5%)	1.01 (0.54; 1.87)	0.98
MAKE 30	20 (21.7%)	17 (18.9%)	37 (20.3%)	0.94 (0.60; 1.50)	0.81
ICU length of stay, day (median, IQR)**	3.00 (1–4)	2.00 (1–4)	2.00 (1–4)	1.07 (0.87; 1.31)	0.53

*p*-values are for comparison between aminoglycosides group and non-aminoglycosides group. Threshold for statistical significance: *p* = 0.05

Reported patient numbers per treatment group represent the Effective Sample Size, rounded to the nearest integer. Categories count may be decimal numbers because of the treatment groups resampling based on a weighting method. Displayed counts are rounded to the closest integer. Proportions are calculated based on the non-rounded values. Due to rounding, the total may differ by ± 1 from the sum using rounded counts from each group

AG, aminoglycosides; CI, confidence interval; ICU, intensive care unit; MAKE 30, Major Adverse Kidney Events at day 30; RRT, renal replacement therapy

*Renal recovery was estimated for the subset of the population that did develop AKI during the course of the ICU (N = 461, and N = 149 after propensity score weighting)

**ICU length of stay was estimated among survivors only (N = 519, and N = 161 after propensity score weighting)

## Discussion

The objective of this multicenter, retrospective study was to evaluate the impact of empiric antibiotic therapy with or without aminoglycosides on ICU mortality in patients with urosepsis. The study included a well-characterized cohort of 580 patients, providing a robust dataset to address this issue. The overall 30-day mortality rate of 10.5% observed in this study is relatively consistent with mortality rates reported in other studies of critically ill patients with urosepsis [4, 5]. Prior to propensity score weighting, there was no difference in mortality between patients who received aminoglycosides and those who did not, even in subgroups such as patients with septic shock, kidney transplant, or infections caused by 3GC-R Enterobacterales. After propensity score weighting, there was a trend towards lower mortality in the aminoglycoside group (7.7% vs. 12.1%), although this difference did not reach statistical significance (aHR = 0.65, *p* = 0.19). This finding raises the possibility that aminoglycosides may be beneficial in a subset of patients. However, further research with adequate statistical power focusing on specific subgroups would be needed to confirm or refute this hypothesis, particularly given the reduction in effective sample size associated with overlap weighting. Furthermore, the similar outcomes between the two groups in terms of renal recovery, need for new RRT and MAKE 30 criteria suggest that aminoglycosides did not have a detrimental impact on renal function.

Current French guidelines, in line with those of American and European societies, recommend the empirical use of a combination of beta-lactams and aminoglycosides for the treatment of urosepsis, particularly in cases of severe infection or septic shock [6–8]. The rationale behind this recommendation is the broad-spectrum coverage provided by the combination therapy and the potential for synergistic effects, which could be particularly valuable in settings with a high prevalence of multidrug-resistant organisms. The Surviving Sepsis Campaign guidelines suggest the use of two antibiotics from different classes in critically ill patients with septic shock to increase the likelihood of appropriate early antimicrobial therapy [37]. Although the theoretical benefits of combination therapy are clear, our findings suggest that in the real-world ICU setting, the routine addition of aminoglycosides may not confer the expected survival benefit. In a Cochrane review, Paul et al*.* found no superiority of combined beta-lactam and aminoglycoside therapy over beta-lactam monotherapy for sepsis and in the subset of patients with urosepsis [38]. Nevertheless, the downward trend in mortality observed after propensity score weighting may suggest a potential benefit of aminoglycosides in a subset of patients. In a meta-analysis of patients with sepsis, Kumar et al*.* showed a survival benefit of aminoglycosides only in the subgroup of patients with septic shock [14]. These findings should encourage further prospective studies to identify patients who may benefit most from aminoglycosides in the management of urosepsis.

The high rates of ESBL-producing Enterobacterales colonization (15.9%) and infection (15.2%) observed on admission in our cohort underscore the challenges of managing urosepsis in the ICU, particularly with the rise of multidrug-resistant organisms. These rates reflect the burden of healthcare-associated infections and the presence of urinary tract devices. Notably, 44.6% of patients with ESBL-producing Enterobacterales colonization developed an ESBL-producing Enterobacterales infection, highlighting the need for careful empirical antibiotic selection. In high-risk cases, the early use of carbapenems may be an alternative to reduce the need for aminoglycosides for spectrum broadening, depending on the ecological context. However, this approach should be re-evaluated based on culture results to allow for de-escalation if possible. Conversely, in low-risk cases, the addition of an aminoglycoside to a narrower-spectrum beta-lactam could provide initial coverage against resistant bacteria and save carbapenems.

Nephrotoxicity is the most significant adverse effect of aminoglycosides with a major prognostic impact in severe infections [20, 25]. Although previous reports, such as those by Llitjos et al., and the Cochrane review by Paul et al., have highlighted an increased risk of acute RRT and higher creatinine levels at ICU discharge associated with aminoglycoside use, our results suggest that aminoglycosides do not necessarily lead to worse renal outcomes [38, 39]. Aminoglycosides were used only as empiric therapy in our cohort, and no patient received an aminoglycoside as definitive therapy, indicating a short course of treatment and potentially reducing the risk of nephrotoxicity. Although specific data on dosage and duration of aminoglycoside treatment were not available, our results suggest that short-term aminoglycoside therapy in critically ill patients does not appear to worsen renal function at day 30.

The strengths of our study include its multicenter design and the use of overlap weighting to control for potential confounders, which increases the robustness of our findings. However, certain limitations must be acknowledged. The retrospective nature of the study inherently limits the ability to draw definitive conclusions and residual confounding cannot be completely excluded despite our efforts to adjust for baseline differences. We acknowledge the limited overlap between treatment groups, which led to a reduced effective sample size after applying overlap weighting; while this improves comparability, it also reduces statistical power, which should be considered when interpreting the results. Furthermore, we were unable to collect detailed data on the frequency and dosage of aminoglycoside administrations, making it impossible to rule out the possibility that some patients received inadequate doses leading potentially to reduced efficacy. Finally, we assessed AKI using only the KDIGO criteria for serum creatinine levels, which may have underestimated its incidence.

## Conclusions

In conclusion, the addition of aminoglycosides to beta-lactam therapy in critically ill patients with urosepsis did not significantly improve 30-day survival. Furthermore, aminoglycosides were not associated with worse renal outcomes. These findings may not be sufficient to challenge the current guidelines advocating the routine use of aminoglycosides in this patient population, but highlight the need for more targeted approaches to antibiotic therapy in the management of urosepsis. Further prospective randomized trials are needed to identify subgroups of patients who may benefit from aminoglycosides.

## Supplementary Information

**Additional file 1.**

## Data Availability

The datasets used and/or analyzed in this study cannot be made public due to data confidentiality regulations, but are available from the corresponding author upon reasonable request.

## Abbreviations

3GC-R: Third-generation cephalosporin-resistant
aHR: Adjusted hazard ratio
AKI: Acute kidney injury
APHP: Assistance Publique-Hôpitaux de Paris
CAI: Community-acquired infections
CHF: Congestive heart failure
CKD: Chronic kidney disease
ESBL: Extended-spectrum beta-lactamase
GFR: Glomerular filtration rate
GNB: Gram-negative bacteria
HAI: Hospital-acquired infections
HCAI: Healthcare-associated infections
HR: Hazard ratio
ICU: Intensive care unit
IPTW: Inverse probability of treatment weighting
IQR: Interquartile
KDIGO: Kidney disease improving global outcomes
MAKE 30: Major adverse kidney events at day 30
RRT: Renal replacement therapy
SAPS II: Simplified Acute physiology score II
SCr: Serum creatinine
SOFA: Sequential organ failure assessment
