# Supplementary material for: Impact of aminoglycosides on survival rate and renal outcomes in patients with urosepsis: a multicenter retrospective study
Source: Ann Intensive Care. 2025 Apr 12;15:52. doi: 10.1186/s13613-025-01469-5 (PMC11992283; doi:10.1186/s13613-025-01469-5)
Supplement: Supplementary file 1 — Additional file 1. [file 13613_2025_1469_MOESM1_ESM.docx]

**Impact of Aminoglycosides on Survival Rate and Renal Outcomes in Patients with Urosepsis : A multicenter Retrospective Study**

**SUPPLEMENTARY MATERIAL**

**Table of contents**

**Additional file 1:** Univariate analysis of mortality factor (page 2)

**Additional file 2:** Standardized Mean Differences in covariates between treatment groups, before and after overlap weighting (page 4)

**Additional file 3:** Propensity Score Validity Assumptions (page 5)

**Additional file 4:** Propensity score distribution according to the treatment group (page 7)

**Additional file 5:** Univariate analysis of baseline patient characteristics (page 8)

**Additional file 6:** Characteristics of patients with sepsis (page 10)

**Additional file 7:** Characteristics of patients with septic shock (page 12)

**Additional file 8 :** Characteristics distribution after applying propensity score overlap weights (page 14)

**Additional file 9:** Unadjusted overall survival with and without aminoglycosides (page 15)

**References** (page 16)

**Additional file 1 :** Univariate analysis of mortality factors

| **Variables** | **Survived**  **(n=519)** | **Died**  **(n=61)** | **Total**  **(n=580)** | ***p*-Value** |
| --- | --- | --- | --- | --- |
| **Age > 65 years** | 310 (59.7%) | 46 (75.4%) | 356 (61.4%) | 0.02 |
| **Male** | 278 (53.6%) | 33 (54.1%) | 311 (53.6%) | 0.94 |
| **Infection onset source** |  |  |  | 0.02 |
| Community-acquired | 192 (37.0%) | 18 (29.5%) | 210 (36.2%) |  |
| Healthcare-associated | 223 (43.0%) | 21 (34.4%) | 244 (42.1%) |  |
| Hospital-acquired | 104 (20.0%) | 22 (36.1%) | 126 (21.7%) |  |
| **Comorbidities** |  |  |  |  |
| Chronic kidney disease | 168 (32.4%) | 27 (44.3%) | 195 (33.6%) | 0.06 |
| Hemodialysis | 10 (1.9%) | 4 (6.6%) | 14 (2.4%) | 0.05 |
| Kidney transplant | 74 (14.3%) | 7 (11.5%) | 81 (14.0%) | 0.55 |
| Indwelling urinary catheter | 61 (11.8%) | 7 (11.5%) | 68 (11.7%) | 0.95 |
| Ureteral stent | 66 (12.7%) | 7 (11.5%) | 73 (12.6%) | 0.78 |
| Nephrostomy | 16 (3.1%) | 1 (1.6%) | 17 (2.9%) | 1.00 |
| Chronic urinary diversion^a^ | 42 (8.1%) | 2 (3.3%) | 44 (7.6%) | 0.18 |
| Previous kidney lithiasis | 48 (9.2%) | 5 (8.2%) | 53 (9.1%) | 0.79 |
| Diabetes | 162 (31.2%) | 21 (34.4%) | 183 (31.6%) | 0.61 |
| Hypertension | 259 (49.9%) | 36 (59.0%) | 295 (50.9%) | 0.18 |
| Congestive heart failure | 59 (11.4%) | 17 (27.9%) | 76 (13.1%) | <0.001 |
| Cirrhosis | 31 (6.0%) | 6 (9.8%) | 37 (6.4%) | 0.24 |
| Previous cancer | 157 (30.3%) | 25 (41.0%) | 182 (31.4%) | 0.09 |
| Active cancer | 87 (16.8%) | 13 (21.3%) | 100 (17.2%) | 0.37 |
| Previous hemopathy | 66 (12.7%) | 7 (11.5%) | 73 (12.6%) | 0.78 |
| Active hemopathy | 53 (10.2%) | 7 (11.5%) | 60 (10.3%) | 0.76 |
| Systemic corticosteroids^b^ | 67 (12.9%) | 5 (8.2%) | 72 (12.4%) | 0.29 |
| Immunosuppressants^c^ | 155 (29.9%) | 18 (29.5%) | 173 (29.8%) | 0.95 |
| HIV | 12 (2.3%) | 1 (1.6%) | 13 (2.2%) | 1.00 |
| Charlson comorbidity index (median, IQR) | 5 (4-7) | 7 (6-9) | 6 (4-8) | <0.001 |
| **Admission characteristics** |  |  |  |  |
| SAPS II score (median, IQR) | 45.00 (35-57) | 64.00 (52-82) | 46.00 (36-60) | <0.001 |
| SOFA score (median, IQR) | 6 (4-8) | 9 (7-12) | 7 (4-9) | <0.001 |
| Septic shock | 288 (55.5%) | 47 (77.0%) | 335 (57.8%) | 0.001 |
| Acute kidney injury | 398 (78.2%) | 50 (87.7%) | 448 (79.2%) | 0.09 |
| Mechanical ventilation | 87 (16.8%) | 28 (45.9%) | 115 (18.8%) | <0.001 |
| Lactate, mmol/L (median, IQR) | 2 (1-3) | 3 (2-7) | 2 (1-3) | <0.001 |
| Neutropenia^d^ | 17 (3.3%) | 3 (4.9%) | 20 (3.4%) | 0.46 |
| Urgent urinary diversion^e^ | 133 (25.6%) | 13 (21.3%) | 146 (25.2%) | 0.46 |
| **Microorganism** |  |  |  |  |
| Enterobacterales | 447 (86.1%) | 50 (82.0%) | 497 (85.7%) | 0.38 |
| *Proteus mirabilis* | 22 (4.2%) | 5 (8.2%) | 27 (4.7%) | 0.19 |
| *Escherichia coli* | 307 (59.2%) | 32 (52.5%) | 339 (58.4%) | 0.32 |
| *Klebsiella spp* (besides *Klebsiella aerogenes*) | 99 (19.1%) | 12 (19.7%) | 111 (19.1%) | 0.91 |
| *Citrobacter koseri* | 11 (2.1%) | 3 (4.9%) | 14 (2.4%) | 0.17 |
| *Klebsiella aerogenes* | 7 (1.3%) | 0 (0.0%) | 7 (1.2%) | 1.00 |
| *Enterobacter cloacae* | 24 (4.6%) | 1 (1.6%) | 25 (4.3%) | 0.50 |
| Other inducible AmpC Enterobacterales^f^ | 18 (3.5%) | 4 (6.6%) | 22 (3.8%) | 0.28 |
| 3GC-R Enterobacterales | 89 (17.1%) | 8 (13.1%) | 97 (16.7%) | 0.43 |
| *Enterococcus faecalis* | 53 (10.2%) | 10 (16.4%) | 63 (10.9%) | 0.14 |
| *Enterococcus faecium* | 13 (2.5%) | 0 (0.0%) | 13 (2.2%) | 0.38 |
| *Staphylococcus aureus* | 9 (1.7%) | 3 (4.9%) | 12 (2.1%) | 0.12 |
| *Pseudomonas aeruginosa* | 43 (8.3%) | 3 (4.9%) | 46 (7.9%) | 0.36 |
| Anaerobic bacteria | 1 (0.2%) | 1 (1.6%) | 2 (0.3%) | 0.20 |
| *Candida spp* | 2 (0.4%) | 1 (1.6%) | 3 (0.5%) | 0.28 |
| Other pathogens | 21 (4.0%) | 5 (8.2%) | 26 (4.5%) | 0.18 |

**Additional file 1 (continued) :** Univariate analysis of mortality factors

| **Variables** | **Survived**  **(n=519)** | **Died**  **(n=61)** | **Total**  **(n=580)** | ***p*-Value** |
| --- | --- | --- | --- | --- |
| Polymicrobial | 84 (16.2%) | 16 (26.2%) | 100 (17.2%) | 0.049 |
| Positive blood culture | 294 (56.6%) | 42 (68.9%) | 336 (57.9%) | 0.07 |
| **Antibiotic therapy** |  |  |  |  |
| 3GC | 197 (38.0%) | 17 (27.9%) | 214 (36.9%) | 0.12 |
| Cefepime | 15 (2.9%) | 1 (1.6%) | 16 (2.8%) | 1.00 |
| Piperacillin-tazobactam | 156 (30.1%) | 20 (32.8%) | 176 (30.3%) | 0.66 |
| Carbapenems^g^ | 153 (29.5%) | 22 (36.1%) | 175 (30.2%) | 0.29 |
| Aminoglycosides | 398 (76.7%) | 46 (75.4%) | 444 (76.6%) | 0.82 |
| Vancomycin | 50 (9.6%) | 8 (13.1%) | 58 (10.0%) | 0.39 |
| Other antibiotics^h^ | 44 (8.5% | 8 (13.1%) | 52 (9.0%) | 0.23 |

Values represent the “number of subjects (%)” unless specified otherwise ; IQR : interquartile range

*p*-Values are for comparison between aminoglycosides group and non-aminoglycosides group. Threshold for statistical significance: *p* = 0.05

HIV: human immunodeficiency virus ; SAPS: Simplified Acute Physiology Score II ; SOFA: Sequential Organ Failure Assessment ; 3GC: third-generation cephalosporins ; 3GC-R: third-generation cephalosporin-resistant

^a^Chronic urinary diversion: ileal conduit, orthotopic neobladder ; ^b^Systemic corticosteroids: ≥10 mg/day chronic prednisone equivalent ; ^c^Immunosuppressants: malignancy or autoimmune-related chemotherapy ; ^d^Neutropenia: neutrophil count of <500 cells/mm3 ; ^e^Urgent urinary diversion: emergency ureteral stenting or nephrostomy ; ^f^Inducible AmpC Enterobacterales: Enterobacterales with inducible chromosomal AmpC beta-lactamases ; ^g^Carbapenems: meropenem or imipenem ; ^h^Other antibiotics: amoxicillin/clavulanic acid, fluoroquinolone, spiramycin, clindamycin, daptomycin, linezolid and metronidazole.

**Additional file 2 :** Standardized Mean Differences in covariates between treatment groups, before and after overlap weighting


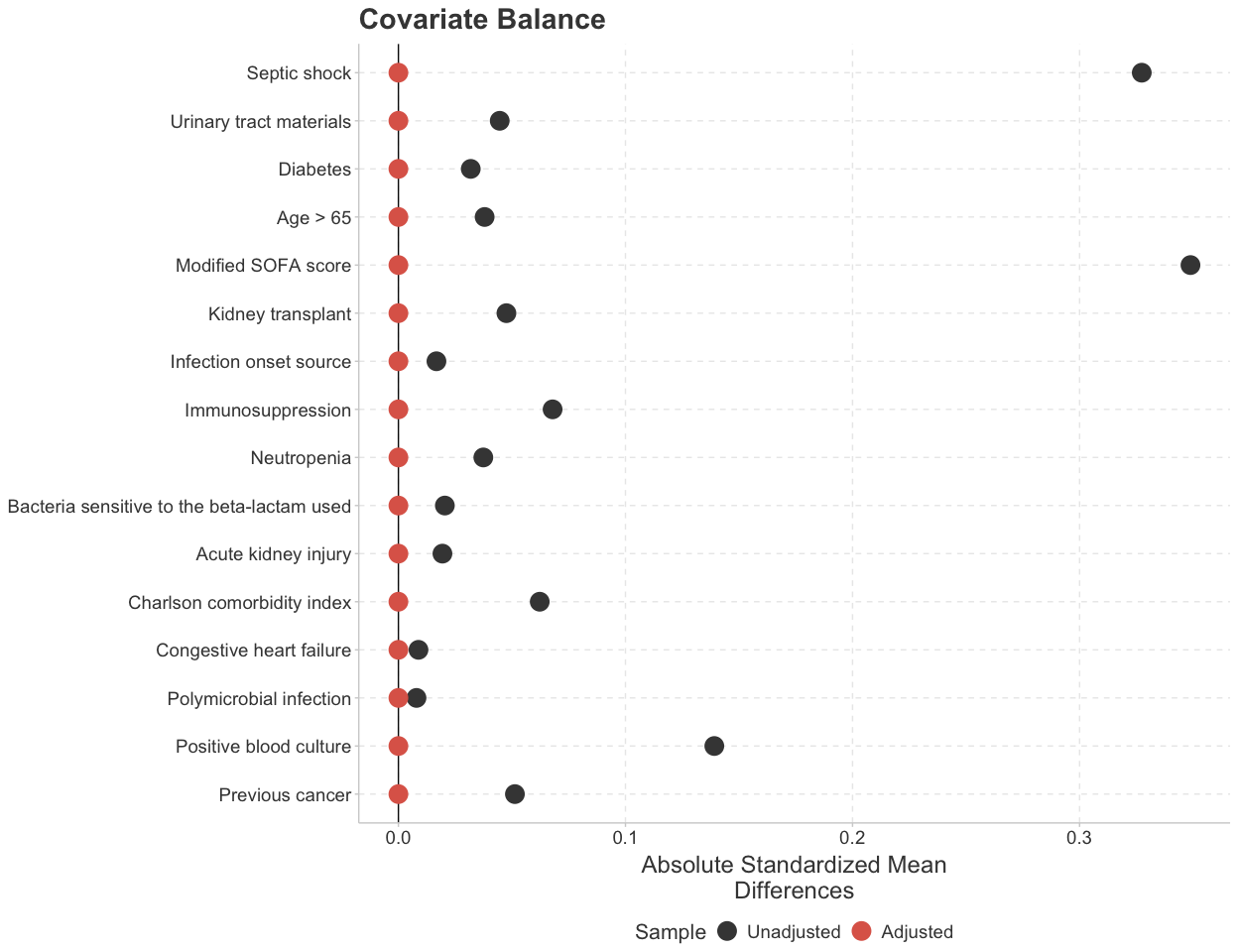


Immunosuppression: malignancy or autoimmune-related chemotherapy, 10 mg/day chronic prednisone equivalent ; Infection onset source: community-acquired *versus* healthcare-associated and hospital-acquired ; Modified SOFA score: SOFA score without cardiovascular and renal criteria ; Neutropenia: neutrophil count of <500 cells/mm3 ; Urinary tract materials: urethral catheter, ureteral stent, nephrostomy

**Additional File 3:** Propensity Score Validity Assumptions

Propensity score model assumptions are generally listed as follows: positivity, consistency, exchangeability, and no misspecification of the propensity score model [1]. We carefully addressed each of these assumptions in our analysis.

- **Exchangeability**, also referred to as ignorability of treatment assignment. This assumption is held if all confounders have been correctly identified and included in the propensity score estimator.

We selected variables for the propensity score model based on domain experts' knowledge, completed with statistical associations. This methodical selection aimed to capture all potential confounding factors. However, confounding adjustment is inherently limited to variables that have been measured. This is a limitation of our study, as it is the case of any observational study. It is usually accepted that this assumption cannot be tested.

- **Positivity**: all patients have a non-zero conditional probability of being assigned to either treatment group.

The distribution of propensity scores demonstrates that no patient has a probability of treatment exposure extremely close to 0 or 1 ( Additional File 8). As this graph shows, no patient has a probability of being exposed to either treatment group close enough to 0 or 1. Potential infringements of this assumption are particularly concerning for methods that might disproportionately emphasize patients with extreme propensity scores (e.g., IPTW) [2]. Overlap weights address this issue, as treated patients are weighted by their probability of not receiving treatment (1 − PS), while untreated patients are weighted by their probability of receiving treatment (PS). These weights naturally attenuate the influence of extreme propensity score, preventing patients with near-certain treatment assignment (PS near 1) or near-certain non-treatment (PS near 0) from dominating the results and reducing precision [3].

- **Consistency**: a given patient potential outcome and observed outcome are equal under the treatment actually received.

This assumption implies no interference between patients in the cohort, meaning that the treatment being given to a patient does not alter the outcome of another patient. While interference could be a critical concern in some studies (such as vaccine efficacy trials where population-level vaccination might impact unvaccinated individuals), it is not a significant issue in the context of our current research.

- **No model misspecification**: the model used to estimate the propensity score has been adequately specified. We chose to model the propensity score using a logistic regression, which happens to be the default choice in propensity score applications in the literature [4]. As with exchangeability, this assumption cannot be formally tested with the data, and requires domain expert knowledge to derive the propensity score model [1].

**Additional File 4:** Propensity score distribution according to the treatment group


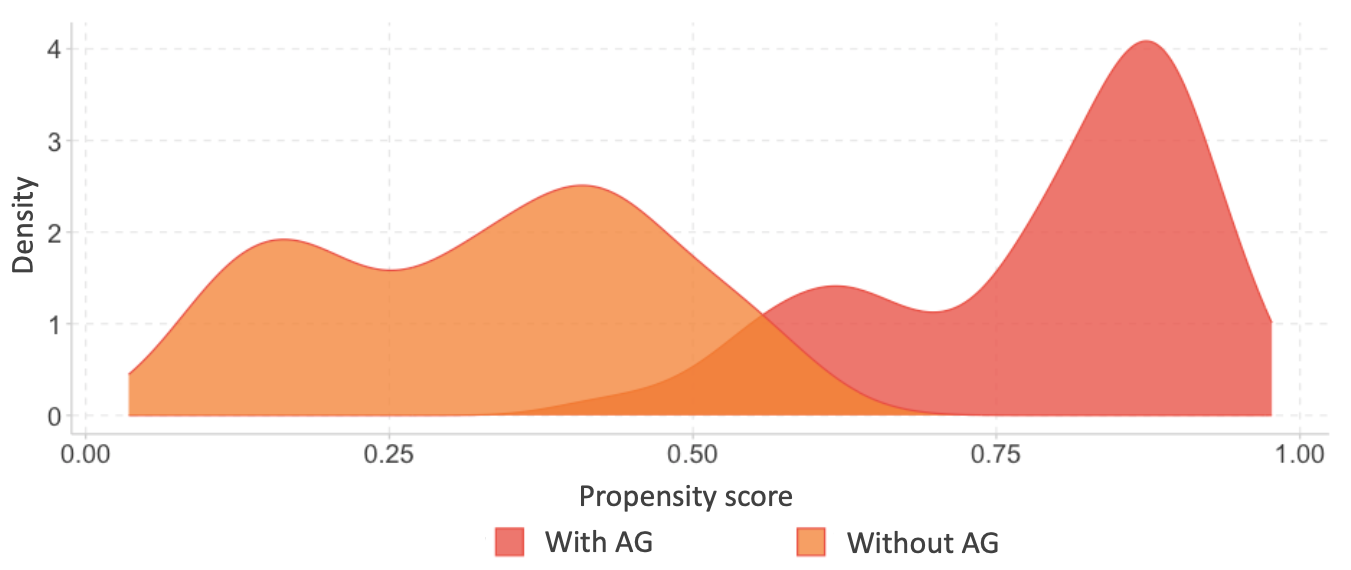


AG: aminoglycosides

**Additional file 5:** Univariate analysis of baseline patient characteristics

| **Variables** | **Without AG**  **(n=136)** | **With AG**  **(n=444)** | **Total**  **(n=580)** | ***p*-Value** |
| --- | --- | --- | --- | --- |
| **Age** (median, IQR) | 69 (58-78) | 69 (58-77) | 69 (58-77) | 0.74 |
| **Male** | 66 (48.5%) | 245 (55.2%) | 311 (53.6%) | 0.17 |
| **Infection onset source** |  |  |  | 0.49 |
| Community-acquired | 49 (36.0%) | 161 (36.3%) | 210 (36.2%) |  |
| Healthcare-associated | 62 (45.6%) | 182 (41.0%) | 244 (42.1%) |  |
| Hospital-acquired | 25 (18.4%) | 101 (22.7%) | 126 (21.7%) |  |
| **Comorbidities** |  |  |  |  |
| Chronic kidney disease | 38 (27.9%) | 157 (35.4%) | 195 (33.6%) | 0.11 |
| Hemodialysis | 0 (0.0%) | 14 (3.2%) | 14 (2.4%) | 0.04 |
| Kidney transplant | 13 (9.6%) | 68 (15.3%) | 81 (14.0%) | 0.09 |
| Uropathy^a^ | 32 (23.5%) | 128 (28.8%) | 160 (27.6%) | 0.23 |
| Diabetes | 39 (28.7%) | 144 (32.4%) | 183 (31.6%) | 0.41 |
| Congestive heart failure | 16 (11.8%) | 60 (13.5%) | 76 (13.1%) | 0.60 |
| Previous malignancy^b^ | 62 (45.6%) | 185 (41.7%) | 247 (42.6%) | 0.42 |
| Immunosuppression^c^ | 37 (27.2%) | 154 (34.7%) | 191 (32.9%) | 0.10 |
| Charlson comorbidity index (median, IQR) | 6 (4-8) | 5.5 (4-8) | 6 (4-8) | 0.73 |
| **Admission characteristics** |  |  |  |  |
| SAPS II score (median, IQR) | 44 (33-54) | 48 (37-61) | 46 (36-60) | 0.01 |
| SOFA score (median, IQR) | 5 (3-7) | 7 (5-10) | 7 (4-9) | <0.001 |
| Septic shock | 44 (32.4%) | 291 (65.5%) | 335 (57.8%) | <0.001 |
| Acute kidney injury | 105 (77.2%) | 343/430 (79.8%) | 448/566 (79.2%) | 0.52 |
| KDIGO stage 1 | 49 (46.7%) | 149 (43.4%) | 198 (44.2%) |  |
| KDIGO stage 2 | 29 (27.6%) | 87 (25.4%) | 116 (25.9%) |  |
| KDIGO stage 3 | 27 (25.7%) | 107 (31.2%) | 134 (29.9%) |  |
| Mechanical ventilation | 17 (12.5%) | 98 (22.1%) | 115 (19.8%) | 0.01 |
| Neutropenia^d^ | 1 (0.7%) | 19 (4.3%) | 20 (3.4%) | 0.048 |
| Urgent urinary diversion^e^ | 34 (25.0%) | 112 (25.2%) | 146 (25.2%) | 0.96 |
| **Microorganism** |  |  |  |  |
| Enterobacterales | 113 (83.1%) | 384 (86.5%) | 497 (85.7%) | 0.32 |
| *Proteus mirabilis* | 11 (8.1%) | 16 (3.6%) | 27 (4.7%) | 0.03 |
| *Escherichia coli* | 78 (57.4%) | 261 (58.8%) | 339 (58.4%) | 0.77 |
| *Klebsiella spp*^f^*/Citrobacter koseri* | 28 (20.6%) | 96 (21.6%) | 124 (21.4%) | 0.80 |
| Inducible AmpC Enterobacterales^g^ | 14 (10.3%) | 38 (8.6%) | 52 (9.0%) | 0.54 |
| 3GC-R Enterobacterales | 22 (16.2%) | 75 (16.9%) | 97 (16.7%) | 0.85 |
| *Enterococcus spp* | 13 (9.6%) | 62 (14.0%) | 75 (12.9%) | 0.18 |
| *Pseudomonas aeruginosa* | 11 (8.1%) | 35 (7.9%) | 46 (7.9%) | 0.94 |
| *Staphylococcus aureus* | 6 (4.4%) | 6 (1.4%) | 12 (2.1%) | 0.03 |
| Other pathogens | 4 (2.9%) | 22 (5.0%) | 26 (4.5%) | 0.32 |
| Polymicrobial | 24 (17.6%) | 76 (17.1%) | 100 (17.2%) | 0.89 |
| Positive blood culture | 64 (47.1%) | 272 (61.3%) | 336 (57.9%) | 0.003 |
| ESBL-producing Enterobacterales colonization/infection in the 6 prior months | 23 (16.9%) | 67 (15.1%) | 90 (15.5%) | 0.61 |
| GNB resistant to the BL used | 12/113 (10.6%) | 28/362 (7.7%) | 40/475 (8.4%) | 0.36 |
| GNB resistant to the AG used | - | 23/325 (7.1%) | - | - |
| GNB susceptible to at least one antibiotic used | 101/113 (89.4%) | 354/362 (97.8%) | 455/475 (95.8%) | <0.001 |
| **Antibiotic therapy** |  |  |  |  |
| 3GC | 51 (37.5%) | 163 (36.7%) | 214 (36.9%) | 0.87 |
| Cefepime | 7 (5.1%) | 9 (2.0%) | 16 (2.8%) | 0.052 |
| Piperacillin-tazobactam | 45 (33.1%) | 131 (29.5%) | 176 (30.3%) | 0.43 |
| Carbapenems^h^ | 31 (22.8%) | 144 (32.4%) | 175 (30.2%) | 0.03 |
| Amikacin | - | 380 (85.6%) | - | - |
| Gentamicin | - | 63 (14.2%) | - | - |
| Vancomycin | 9 (6.6%) | 49 (11.0%) | 58 (10.0%) | 0.13 |
| Other antibiotics^i^ | 19 (14.0%) | 33 (7.4%) | 52 (9.0%) | 0.02 |

Values represent the “number of subjects (%)” except as noted ; IQR : interquartile range. *p*-Values are for comparison between aminoglycosides group and non-aminoglycosides group. Threshold for statistical significance: *p* = 0.05

Missing data were present only for “GNB resistant to the BL used”, “GNB resistant to the AG used”, and “GNB susceptible to at least one antibiotic used” with denominators added in table cells when necessary to indicate the actual number available observations.

AG: aminoglycosides ; BL: beta-lactam ; ESBL: extended spectrum beta-lactamase ; GNB: Gram-negative bacteria ; KDIGO: Kidney Disease Improving Global Outcomes ; SAPS: Simplified Acute Physiology Score II ; SOFA: Sequential Organ Failure Assessment ; 3GC: third-generation cephalosporins ; 3GC-R: third generation cephalosporin-resistant

^a^Uropathy: urethral catheter, ureteral stent, nephrostomy, ileal conduit, orthotopic neobladder ; ^b^Previous malignancy: previous cancer or hemopathy ; ^c^Immunosuppression: malignancy or autoimmune-related chemotherapy, ≥10 mg/day chronic prednisone equivalent ; ^d^Neutropenia: neutrophil count of <500 cells/mm3 ; ^e^Urgent urinary diversion: emergency ureteral stenting or nephrostomy ; ^f^Except *Klebsiella aerogenes* ; ^g^Inducible AmpC Enterobacterales: Enterobacterales with inducible chromosomal AmpC beta-lactamases ; ^h^Carbapenems: meropenem or imipenem ; ^i^Other antibiotics: amoxicillin/clavulanic acid (one patient), fluoroquinolone, spiramycin, clindamycin, daptomycin, linezolid and metronidazole.

**Additional file 6 :** Characteristics of patients with sepsis

| **Variables** | **Without AG**  **(n=92)** | **With AG**  **(n=153)** | **Total**  **(n=245)** | ***p*-Value** |
| --- | --- | --- | --- | --- |
| **Age** (median, IQR) | 68 (58-75) | 64 (53-76) | 67 (55-75) | 0.33 |
| **Male** | 49 (53.3%) | 90 (58.8%) | 139 (56.7%) | 0.40 |
| **Infection onset source** |  |  |  | 0.47 |
| Community-acquired | 33 (35.9%) | 58 (37.9%) | 91 (37.1%) |  |
| Healthcare-associated | 45 (48.9%) | 64 (41.8%) | 109 (44.5%) |  |
| Hospital-acquired | 14 (15.2%) | 31 (20.3%) | 45 (18.4%) |  |
| **Comorbidities** |  |  |  |  |
| Chronic kidney disease | 28 (30.4%) | 50 (32.7%) | 78 (31.8%) | 0.72 |
| Hemodialysis | 0 (0.0%) | 4 (2.6%) | 4 (1.6%) | 0.12 |
| Kidney transplant | 10 (10.9%) | 28 (18.3%) | 38 (15.5%) | 0.12 |
| Uropathy^a^ | 21 (22.8%) | 41 (26.8%) | 62 (25.3%) | 0.49 |
| Diabetes | 23 (25.0%) | 43 (28.1%) | 66 (26.9%) | 0.60 |
| Congestive heart failure | 10 (10.9%) | 17 (11.1%) | 27 (11.0%) | 0.95 |
| Previous malignancy^b^ | 44 (47.8%) | 60 (39.2%) | 104 (42.4%) | 0.19 |
| Immunosuppression^c^ | 25 (27.2%) | 53 (34.6%) | 78 (31.8%) | 0.22 |
| Charlson comorbidity index (median, IQR) | 6 (4-8) | 5 (3-7) | 5 (3-8) | 0.27 |
| **Admission characteristics** |  |  |  |  |
| SAPS II score (median, IQR) | 37 (30-48) | 37 (29-47) | 37 (30-48) | 0.35 |
| SOFA score (median, IQR) | 4 (2-5) | 4 (3-6) | 4 (3-5) | 0.16 |
| Acute kidney injury | 69 (75.0%) | 104/149 (69.8%) | 173/241 (71.8%) | 0.38 |
| KDIGO stage 1 | 35 (50.7%) | 53 (51.0%) | 88 (50.9%) |  |
| KDIGO stage 2 | 14 (20.3%) | 23 (22.1%) | 37 (21.4%) |  |
| KDIGO stage 3 | 20 (29.0%) | 28 (26.9%) | 48 (27.7%) |  |
| Mechanical ventilation | 5 (5.4%) | 1 (0.7%) | 6 (2.4%) | 0.02 |
| Neutropenia^d^ | 0 (0.0%) | 3 (2.0%) | 3 (1.2%) | 0.18 |
| Urgent urinary diversion^e^ | 16 (17.4%) | 18 (11.8%) | 34 (13.9%) | 0.22 |
| **Microorganism** |  |  |  |  |
| Enterobacterales | 78 (84.8%) | 132 (86.3%) | 210 (85.7%) | 0.75 |
| *Proteus mirabilis* | 7 (7.6%) | 1 (0.7%) | 8 (3.3%) | 0.003 |
| *Escherichia coli* | 53 (57.6%) | 94 (61.4%) | 147 (60.0%) | 0.55 |
| *Klebsiella spp*^f^*/Citrobacter koseri* | 19 (20.7%) | 32 (20.9%) | 51 (20.8%) | 0.96 |
| Inducible AmpC Enterobacterales^g^ | 10 (10.9%) | 13 (8.5%) | 23 (9.4%) | 0.54 |
| 3GC-R Enterobacterales | 14 (15.2%) | 25 (16.3%) | 39 (15.9%) | 0.82 |
| *Enterococcus spp* | 12 (13.0%) | 16 (10.5%) | 28 (11.4%) | 0.54 |
| *Pseudomonas aeruginosa* | 7 (7.6%) | 8 (5.2%) | 15 (6.1%) | 0.45 |
| *Staphylococcus aureus* | 2 (2.2%) | 5 (3.3%) | 7 (2.9%) | 0.62 |
| Other pathogens | 2 (2.2%) | 9 (5.9%) | 11 (4.5%) | 0.18 |
| Polymicrobial | 16 (17.4%) | 20 (13.1%) | 36 (14.7%) | 0.36 |
| Positive blood culture | 42 (45.7%) | 79 (51.6%) | 121 (49.4%) | 0.36 |
| ESBL-producing Enterobacterales colonization/infection in the 6 prior months | 18 (19.6%) | 19 (12.4%) | 37 (15.1%) | 0.13 |
| GNB resistant to the BL used | 8/76 (10.5%) | 9/129 (7.0%) | 17/205 (8.3%) | 0.37 |
| GNB resistant to the AG used | - | 7/118 (6.1%) | - | - |
| GNB susceptible to at least one antibiotic used | 68/76 (89.5%) | 127/129 (98.4%) | 195/205 (95.1%) | 0.004 |
| **Antibiotic therapy** |  |  |  |  |
| 3GC | 41 (44.6%) | 76 (49.7%) | 117 (47.8%) | 0.44 |
| Cefepime | 5 (5.4%) | 4 (2.6%) | 9 (3.7%) | 0.26 |
| Piperacillin-tazobactam | 26 (28.3%) | 36 (23.5%) | 62 (25.3%) | 0.41 |
| Carbapenems^h^ | 20 (21.7%) | 39 (25.5%) | 59 (24.1%) | 0.51 |
| Amikacin | - | 122 (79.7%) | - | - |
| Gentamicin | - | 30 (19.6%) | - | - |
| Vancomycin | 2 (2.2%) | 13 (8.5%) | 15 (6.1%) | 0.046 |
| Other antibiotics | 7 (7.6%) | 15 (9.8%) | 22 (9.0%) | 0.56 |
| **Mortality** | 7 (7.6%) | 7 (4.6%) | 14 (5.7%) | 0.32 |

Values represent the “number of subjects (%)” except as noted ; IQR : interquartile range

*p*-Values are for comparison between aminoglycosides group and non-aminoglycosides group. Threshold for statistical significance: *p* = 0.05

AG: aminoglycosides ; BL: beta-lactam ; ESBL: extended spectrum beta-lactamase ; GNB: Gram-negative bacteria ; KDIGO: Kidney Disease Improving Global Outcomes ; SAPS: Simplified Acute Physiology Score II ; SOFA: Sequential Organ Failure Assessment ; 3GC: third-generation cephalosporins ; 3GC-R : third-generation cephalosporin-resistant

^a^Uropathy: urethral catheter, ureteral stent, nephrostomy, ileal conduit, orthotopic neobladder ; ^b^Previous malignancy: previous cancer or hemopathy ; ^c^Immunosuppression: malignancy or autoimmune-related chemotherapy, ≥10 mg/day chronic prednisone equivalent ; ^d^Neutropenia: neutrophil count of <500 cells/mm3 ; ^e^Urgent urinary diversion: emergency ureteral stenting or nephrostomy ; ^f^Except *Klebsiella aerogenes* ; ^g^Inducible AmpC Enterobacterales: Enterobacterales with inducible chromosomal AmpC beta-lactamases ; ^h^Carbapenems: meropenem or imipenem

**Additional file 7:** Characteristics of patients with septic shock

| **Variables** | **Without AG**  **(n=44)** | **With AG**  **(n=291)** | **Total**  **(n=335)** | ***p*-Value** |
| --- | --- | --- | --- | --- |
| **Age** (median, IQR) | 73 (63-81) | 71 (60-77) | 71 (60-78) | 0.39 |
| **Male** | 17 (38.6%) | 155 (53.3%) | 172 (51.3%) | 0.07 |
| **Infection onset source** |  |  |  | 0.97 |
| Community-acquired | 16 (36.4%) | 103 (35.4%) | 119 (35.5%) |  |
| Healthcare-associated | 17 (38.6%) | 118 (40.5%) | 135 (40.3%) |  |
| Hospital-acquired | 11 (25.0%) | 70 (24.1%) | 81 (24.2%) |  |
| **Comorbidities** |  |  |  |  |
| Chronic kidney disease | 10 (22.7%) | 107 (36.8%) | 117 (34.9%) | 0.07 |
| Hemodialysis | 0 (0.0%) | 10 (3.4%) | 10 (3.0%) | 0.21 |
| Kidney transplant | 3 (6.8%) | 40 (13.7%) | 43 (12.8%) | 0.20 |
| Uropathy^a^ | 11 (25.0%) | 87 (29.9%) | 98 (29.3%) | 0.51 |
| Diabetes | 16 (36.4%) | 101 (34.7%) | 117 (34.9%) | 0.83 |
| Congestive heart failure | 6 (13.6%) | 43 (14.8%) | 49 (14.6%) | 0.84 |
| Previous malignancy^b^ | 18 (40.9%) | 125 (43.0%) | 143 (42.7%) | 0.80 |
| Immunosuppression^c^ | 12 (27.3%) | 101 (34.7%) | 113 (33.7%) | 0.33 |
| Charlson comorbidity index (median, IQR) | 6 (5-7) | 6 (4-8) | 6 (4-8) | 0.80 |
| **Admission characteristics** |  |  |  |  |
| SAPS II score (median, IQR) | 52 (45-70) | 54 (43-67) | 54 (43-68) | 0.72 |
| SOFA score (median, IQR) | 7 (6-9) | 8 (7-11) | 8 (7-11) | 0.03 |
| Acute kidney injury | 36 (81.8%) | 239/281 (85.1%) | 275/325 (84.6%) | 0.58 |
| KDIGO stage 1 | 14 (38.9%) | 96 (40.2%) | 110 (40.0%) |  |
| KDIGO stage 2 | 15 (41.7%) | 64 (26.8%) | 79 (28.7%) |  |
| KDIGO stage 3 | 7 (19.4%) | 79 (33.1%) | 86 (31.3%) |  |
| Mechanical ventilation | 12 (27.3%) | 97 (33.3%) | 109 (32.5%) | 0.42 |
| Neutropenia^d^ | 1 (2.3%) | 16 (5.5%) | 17 (5.1%) | 0.36 |
| Urgent urinary diversion^e^ | 18 (40.9%) | 94 (32.3%) | 112 (33.4%) | 0.26 |
| **Microorganism** |  |  |  |  |
| Enterobacterales | 35 (79.5%) | 252 (86.6%) | 287 (85.7%) | 0.21 |
| *Proteus mirabilis* | 4 (9.1%) | 15 (5.2%) | 19 (5.7%) | 0.29 |
| *Escherichia coli* | 25 (56.8%) | 167 (57.4%) | 192 (57.3%) | 0.94 |
| *Klebsiella spp*^f^*/Citrobacter koseri* | 9 (20.5%) | 64 (22.0%) | 73 (21.8%) | 0.82 |
| Inducible AmpC Enterobacterales^g^ | 4 (9.1%) | 25 (8.6%) | 29 (8.7%) | 0.91 |
| 3GC-R Enterobacterales | 8 (18.2%) | 50 (17.2%) | 58 (17.3%) | 0.87 |
| *Enterococcus spp* | 1 (2.3%) | 46 (15.8%) | 47 (14.0%) | 0.02 |
| *Pseudomonas aeruginosa* | 4 (9.1%) | 27 (9.3%) | 31 (9.3%) | 0.97 |
| *Staphylococcus aureus* | 4 (9.1%) | 1 (0.3%) | 5 (1.5%) | <0.001 |
| Other pathogens | 2 (4.5%) | 13 (4.5%) | 15 (4.5%) | 0.98 |
| Polymicrobial | 8 (18.2%) | 56 (19.2%) | 64 (19.1%) | 0.87 |
| Positive blood culture | 22 (50.0%) | 193 (66.3%) | 215 (64.2%) | 0.04 |
| ESBL-producing Enterobacterales colonization/infection in the 6 prior months | 5 (11.4%) | 48 (16.5%) | 53 (15.8%) | 0.39 |
| GNB resistant to the BL used | 4/36 (10.8%) | 19/233 (8.2%) | 23/269 (8.5%) | 0.59 |
| GNB resistant to the AG used | - | 16/211 (7.6%) | - | - |
| GNB susceptible to at least one antibiotic used | 33/37 (89.2%) | 227/233 (97.4%) | 260/270 (96.3%) | 0.014 |
| **Antibiotic therapy** |  |  |  |  |
| 3GC | 10 (22.7%) | 87 (29.9%) | 97 (29.0%) | 0.33 |
| Cefepime | 2 (4.5%) | 5 (1.7%) | 7 (2.1%) | 0.22 |
| Piperacillin-tazobactam | 19 (43.2%) | 95 (32.6%) | 114 (34.0%) | 0.17 |
| Carbapenems^h^ | 11 (25.0%) | 105 (36.1%) | 116 (34.6%) | 0.15 |
| Amikacin | - | 258 (88.7%) | - | - |
| Gentamicin | - | 33 (11.3%) | - | - |
| Vancomycin | 7 (15.9%) | 36 (12.4%) | 43 (12.8%) | 0.51 |
| Other antibiotics | 9 (20.5%) | 16 (5.5%) | 25 (7.5%) | <0.001 |
| **Mortality** | 8 (18.2%) | 39 (13.4%) | 47 (14.0%) | 0.40 |

Values represent the “number of subjects (%)” except as noted ; IQR : interquartile range

*p*-Values are for comparison between aminoglycosides group and non-aminoglycosides group. Threshold for statistical significance: *p* = 0.05

AG: aminoglycosides ; BL: beta-lactam ; ESBL: extended spectrum beta-lactamase ; GNB: Gram-negative bacteria ; KDIGO: Kidney Disease Improving Global Outcomes ; SAPS: Simplified Acute Physiology Score II ; SOFA: Sequential Organ Failure Assessment ; 3GC: third-generation cephalosporins ; 3GC-R: third-generation cephalosporin-resistant

^a^Uropathy: urethral catheter, ureteral stent, nephrostomy, ileal conduit, orthotopic neobladder ; ^b^Previous malignancy: previous cancer or hemopathy ; ^c^Immunosuppression: malignancy or autoimmune-related chemotherapy, ≥10 mg/day chronic prednisone equivalent ; ^d^Neutropenia: neutrophil count of <500 cells/mm3 ; ^e^Urgent urinary diversion: emergency ureteral stenting or nephrostomy ; ^f^Except *Klebsiella aerogenes* ; ^g^Inducible AmpC Enterobacterales: Enterobacterales with inducible chromosomal AmpC beta-lactamases ; ^h^Carbapenems: meropenem or imipenem

**Additional file 8:** Characteristics distribution after applying propensity score overlap weights

| **Variables** | **Without AG**  **(n=91)** | **With AG**  **(n=91)** | **Total**  **(n=182)** |
| --- | --- | --- | --- |
| Septic shock | 37 (40.9%) | 37 (40.9%) | 74 (40.9%) |
| Urinary tract materials^a^ | 21 (23.0%) | 21 (23.0%) | 42 (23.0%) |
| Diabetes | 28 (30.4%) | 28 (30.4%) | 55 (30.4%) |
| Age > 65 years | 22 (24.5%) | 22 (24.5%) | 44 (24.5%) |
| Modified SOFA score (median, IQR)^b^ | 2.00 (0-3) | 2.00 (1-3) | 2.00 (1-3) |
| Kidney transplant | 10 (10.8%) | 10 (10.8%) | 20 (10.8%) |
| Infection onset source^c^ | 57 (62.8%) | 57 (62.8%) | 114 (62.8%) |
| Immunosuppression^d^ | 26 (28.9%) | 26 (28.9%) | 53 (28.9%) |
| Neutropenia^e^ | 1 (1.1%) | 1 (1.1%) | 2 (1.1%) |
| Bacteria sensitive to the beta-lactam used | 78 (86.2%) | 78 (86.2%) | 156 (86.2%) |
| Acute kidney injury | 71 (78.4%) | 71 (78.4%) | 142 (78.4%) |
| Charlson comorbidity index (median, IQR) | 6.00 (4-7) | 5.00 (4-8) | 6.00 (4-8) |
| Congestive heart failure | 11 (12.1%) | 11 (12.1%) | 22 (12.1%) |
| Polymicrobial infection | 16 (17.2%) | 16 (17.2%) | 31 (17.2%) |
| Positive blood culture | 46 (50.5%) | 46 (50.5%) | 92 (50.5%) |
| Previous cancer | 31 (34.1%) | 31 (34.1%) | 62 (34.1%) |

Values represent the “number of subjects (%)” unless specified otherwise. IQR : interquartile range

Reported patient numbers per treatment group represent the Effective Sample Size, rounded to the nearest integer. Categories count may be decimal numbers because of the treatment groups resampling based on a weighting method. Displayed counts are rounded to the closest integer. Proportions are calculated based on the non-rounded values. Due to rounding, the total may differ by ± 1 from the sum using rounded counts from each group.

^a^Urinary tract materials: urethral catheter, ureteral stent, nephrostomy ; ^b^Modified SOFA score: SOFA score without cardiovascular and renal criteria ; ^c^Infection onset source: community-acquired *versus* healthcare-associated and hospital-acquired ; ^d^Immunosuppression: malignancy or autoimmune-related chemotherapy, 10 mg/day chronic prednisone equivalent ; ^e^Neutropenia: neutrophil count of <500 cells/mm3

**Additional file 9:** Unadjusted overall survival with and without aminoglycosides

Kaplan–Meier curves showing the probability of survival according to the empirical antibiotherapy group (treatment with aminoglycosides (AG) compared to treatment without AG)

**References**

1. Austin PC, Stuart EA (2015) Moving towards best practice when using inverse probability of treatment weighting (IPTW) using the propensity score to estimate causal treatment effects in observational studies. Stat Med 34:3661–3679. https://doi.org/10.1002/sim.6607

2. Hajage D, Tubach F, Steg PG, et al (2016) On the use of propensity scores in case of rare exposure. BMC Med Res Methodol 16:38. https://doi.org/10.1186/s12874-016-0135-1

3. Thomas LE, Li F, Pencina MJ (2020) Overlap Weighting: A Propensity Score Method That Mimics Attributes of a Randomized Clinical Trial. JAMA 323:2417. https://doi.org/10.1001/jama.2020.7819

4. Chattopadhyay A, Hase CH, Zubizarreta JR (2020) Balancing vs modeling approaches to weighting in practice. Statistics in Medicine 39:3227–3254. https://doi.org/10.1002/sim.8659
